# Comparison of percutaneous and open repair of Achilles tendon rupture: results and complications from a single institution

**DOI:** 10.1186/s12893-024-02333-2

**Published:** 2024-02-06

**Authors:** Fabijan Čukelj jr, Dejan Blažević, Fabijan Čukelj, Srećko Sabalić, Ivan Benčić, Tomislav Ćuti, Dinko Pivalica, Bore Bakota, Dinko Vidović

**Affiliations:** 1 School of Medicine, University of Zagreb, Šalata 3, Zagreb, 10000 Croatia; 2 Department of Traumatology, Sestre milosrdnice University Hospital Center, Draškovićeva 19, Zagreb, 10000 Croatia; 3 University of Applied Health Sciences, Mlinarska cesta 38, Zagreb, 10000 Croatia; 4 School of Medicine, Catholic University of Croatia, Ilica 242, Zagreb, 10000 Croatia; 5 School of Medicine, University of Split, Šoltanska 2, Split, 21000 Croatia; 6 Department of Physical Medicine and Rehabilitation with Rheumatology, University Hospital of Split, Spinčićeva 1, Split, 21000 Croatia; 7 Tauern Klinikum, Paracelsusstrasse 8, Zell am See, 5700 Austria; 8 MedUni Klinikum LKH, Auenbruggerplatz 15, Graz, 8036 Austria; 9 School of Dental Medicine, University of Zagreb, Gundulićeva 5, Zagreb, 10000 Croatia

**Keywords:** Achilles tendon, Achilles tendon rupture, Open repair, Percutaneous repair

## Abstract

**Background:**

The Achilles tendon is the strongest tendon in the human body, but it is prone to injury, especially in modern times when recreational sports are growing in popularity. As a result, Achilles tendon rupture is becoming an increasingly common medical problem in modern society. The main objective of this study was to compare the outcomes of percutaneous repair and open repair for the treatment of Achilles tendon rupture.

**Methods:**

A retrospective study was conducted involving a total of 316 patients who had undergone surgical treatment for Achilles tendon rupture between 2013 and 2021. The data collected from the medical history of these patients included the type of surgical procedure, the mechanism of injury, the age and sex of the patients, the time spent in the hospital, and any possible complications of the surgical treatment (such as infections, reruptures, or sural nerve injuries).

**Results:**

The study revealed that there was no significant difference between percutaneous and open surgical approaches in terms of sural nerve injury. However, there was a statistically significant advantage of the percutaneous method in terms of the number of infections, which was significantly lower than that of the open method. Additionally, the median length of hospital stay was found to be four days longer with the open approach. However, the study noted that a statistically significant advantage of the percutaneous method for rerupture could not be established due to the small number of patients with rerupture and the insufficient ratio of patients with rerupture in relation to the size of the observed population.

**Conclusions:**

Percutaneous repair is an effective treatment option for Achilles tendon rupture and has outcomes equal to or better than those of open repair. Therefore, this approach is recommended as the preferred method of treatment due to the presence of fewer complications, provided that the indications for this technique are appropriate.

## Background

The Achilles tendon, which is now a well-known eponym for tendo calcanei, is the thickest and strongest tendon in the human body. It is considered a crucial feature of human anatomy and is thought to have played a significant role in human evolution [1]. The ability to run has been critically linked to natural selection, and humans’ unique combination of speed and exceptional endurance has been underestimated. The Achilles tendon is one of the key factors involved in this development [2]. Despite its importance, the Achilles tendon is vulnerable to injury, and in modern times, with the growing popularity of recreational sports, rupture of the Achilles tendon has become one of the more frequent medical problems in today’s society, particularly in middle-aged men [3]. Dysmetabolic conditions, including thyroid dysfunctions, high cholesterol levels, diabetes mellitus, and obesity, may contribute to the development of Achilles tendinopathy. This condition is a significant predisposing factor for rupture of the Achilles tendon [4].

Another issue is the delayed diagnosis of acute ruptures, where more than 20% of such injuries are not recognized in time or patients do not seek emergency medical help, underestimating the severity of the injury [5]. Acute ruptures of the Achilles tendon are treated with various approaches, including conservative and surgical approaches [6]. Although there is a general consensus that the best approach is surgery, despite extensive research in the last decade, the decision for the best treatment protocol for these ruptures remains a topic of debate and disagreement [7].

The aim of this study was to determine the success of percutaneous repair for the treatment of Achilles tendon rupture by comparing primary treatment outcomes (incidence of postoperative wound infection, rerupture, sural nerve injury, days of hospitalization) with those recorded during open repair.

## Methods

To conduct this research, data were collected from patients who underwent surgical treatment for ruptured Achilles tendons at the Department of Traumatology, Sestre milosrdnice University Hospital Center in Zagreb, Croatia, from 2013 to 2021. Prior to conducting the research, ethical approval was obtained from the Sestre milosrdnice University Hospital Center Ethical Commission (ethical approval number: 003–06/22 − 03/011) to access the medical histories of qualified patients. The archives and protocols used were searched to collect data on the number of patients, surgical methods used for treating Achilles tendon rupture, complications of surgical treatment (such as postoperative infection of the surgical wound, Achilles tendon rerupture, and sural nerve injury), and hospitalization time from admission to discharge. Basic patient data, including sex, age at the time of the operation, mechanism of injury, leg injury (left or right), and location of rupture (distance from tendon insertion on the calcaneus in centimeters), were also collected.

**Fig. 1:**
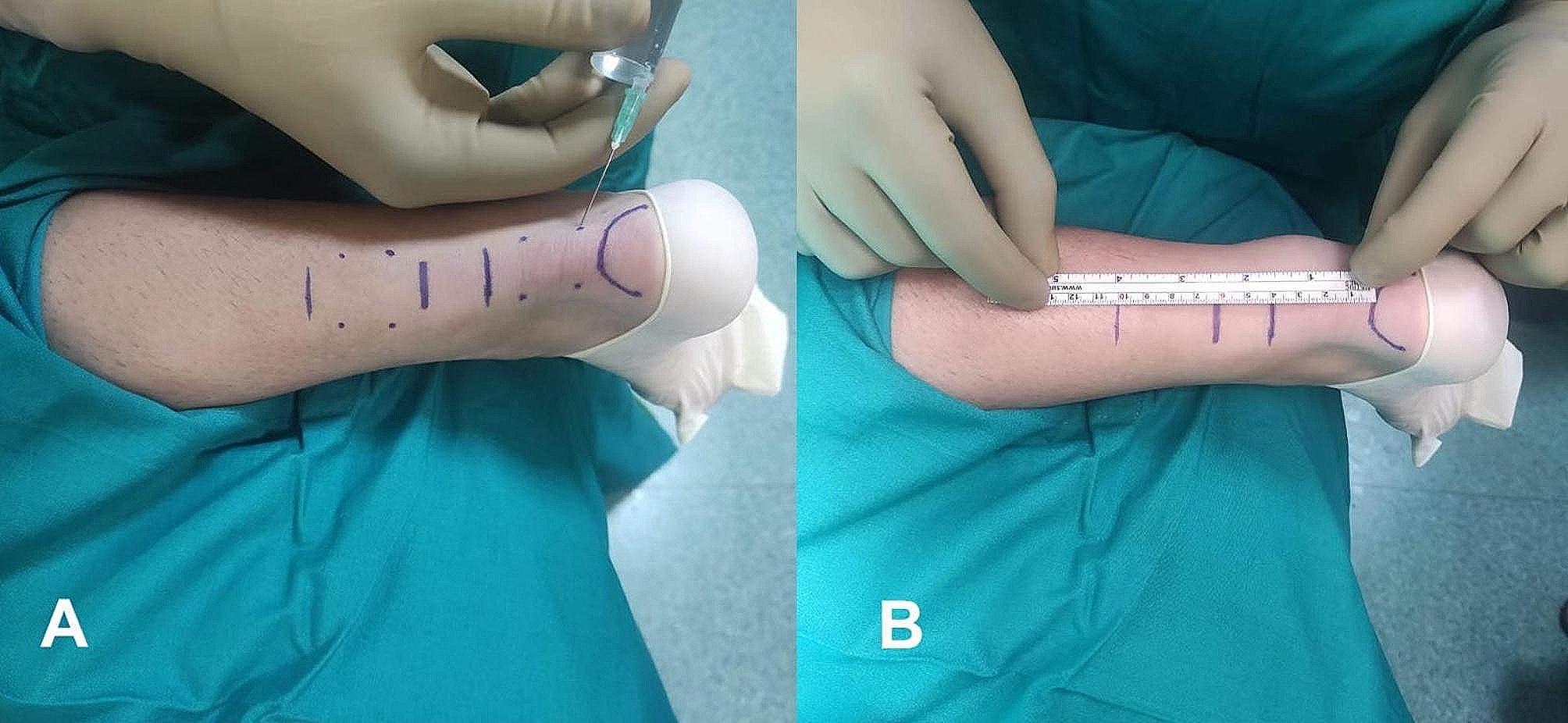
Percutaneous technique for Achilles tendon repair. (**A**) Lidocaine application. (**B**) Visualization of the sural nerve path and infiltration sites

The surgical technique used in this study was based on a method developed by Čretnik et al. [7]. Patients were in a supine position with the injured foot in plantar flexion of approximately 25° and received local anaesthesia without a tourniquet. Before the procedure, the rupture and site of diastasis were localized. Using 15–20 mL of 1% plain lidocaine, the skin and subcutaneous tissue were infiltrated 5 cm proximal to the palpable gap and 4 cm distal through 8 incisions (Fig. 1A). No other drugs, nerve blocks, or anaesthetics were needed. Special attention was given to the lateral side of the Achilles complex, particularly the proximal part, where the sural nerve is located nearby and crosses the Achilles tendon medially. The site of infiltration did not exceed the edge of the Achilles tendon in this area (Fig. 1B). Patients were warned of any change or pain in the area of the sural nerve during puncture or infiltration. If necessary, the injection site was moved 0.5 to 1 cm medially. A resorbable suture was recommended to reduce symptoms over time if a nerve injury occurred. In this study, Vicryl suture 2 w was utilized for this purpose.

**Fig. 2:**
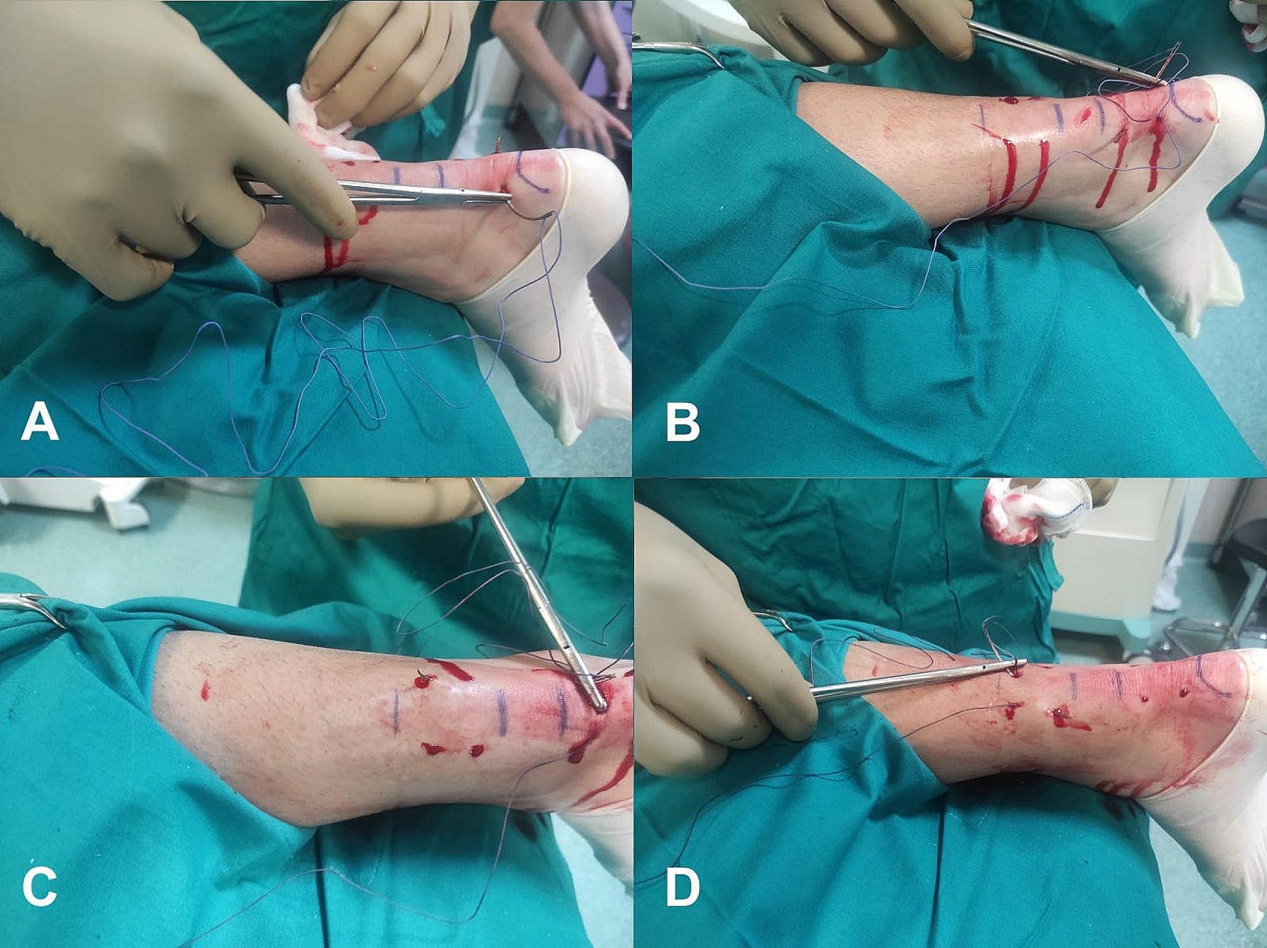
Percutaneous technique for Achilles tendon repair. (**A**) The suture was inserted through the most distal medial incision, and it was directed toward the corresponding distal lateral incision. (**B**) The suture was cross-passed from the medial side to the lateral side utilizing the distal incisions closest to the rupture. (**C**) The suture on the lateral side was then threaded through the most proximal lateral incision. (**D**) The suture was again cross-passed from the lateral to the medial side through the proximal incisions nearest to the rupture

**Fig. 3:**
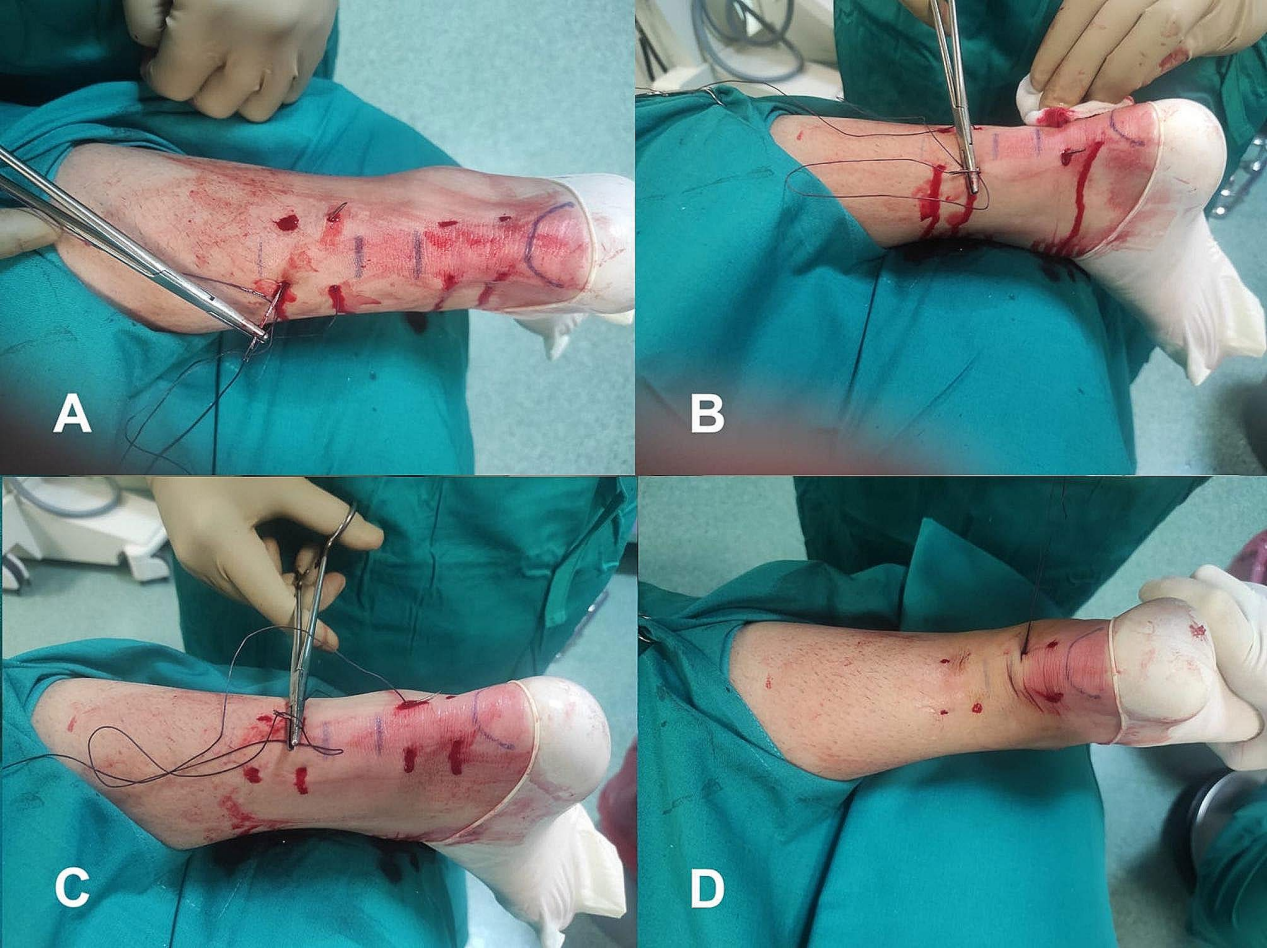
Percutaneous technique for Achilles tendon repair. (**A**) The suture was cross-passed from the medial to the lateral side through the proximal incisions nearest to the rupture. (**B**) The medial suture was threaded through the third distal incision on the medial side, after which the incision was extended to the same level as the corresponding lateral incision. (**C**) The lateral suture was looped through the third proximal incision on the lateral side. (**D**) Both suture limbs were threaded through the third distal incision on the lateral side, culminating in tightening of the knot

In the percutaneous technique, eight incisions of 3–5 mm each were employed. These incisions were strategically positioned around the Achilles tendon rupture: four proximally and four distally. For each set of four incisions, two were made medial to the Achilles tendon, and two were made lateral to it. The suturing process began by inserting the suture through the most distal medial incision and directing it toward the corresponding distal lateral incision (Fig. 2A). Subsequently, the suture was cross-passed from the medial side to the lateral side utilizing the distal incisions closest to the rupture (Fig. 2B). After continuing the process, the suture on the lateral side was then threaded through the most proximal lateral incision (Fig. 2C). A similar procedure was replicated on the medial side. Following this, the suture was again cross-passed, this time from the lateral to the medial side, through the proximal incisions nearest to the rupture (Fig. 2D). The same steps were followed for the medial side (Fig. 3A). The final stage involved threading the medial suture back through the third distal incision on the medial side (Fig. 3B) and then extending it to a corresponding lateral incision at the same level. Simultaneously, the lateral suture was looped through the third proximal incision on the lateral side (Fig. 3C). This arrangement led to both suture limbs being threaded through the third distal incision on the lateral side, culminating in tightening of the knot (Fig. 3D).

**Fig. 4:**
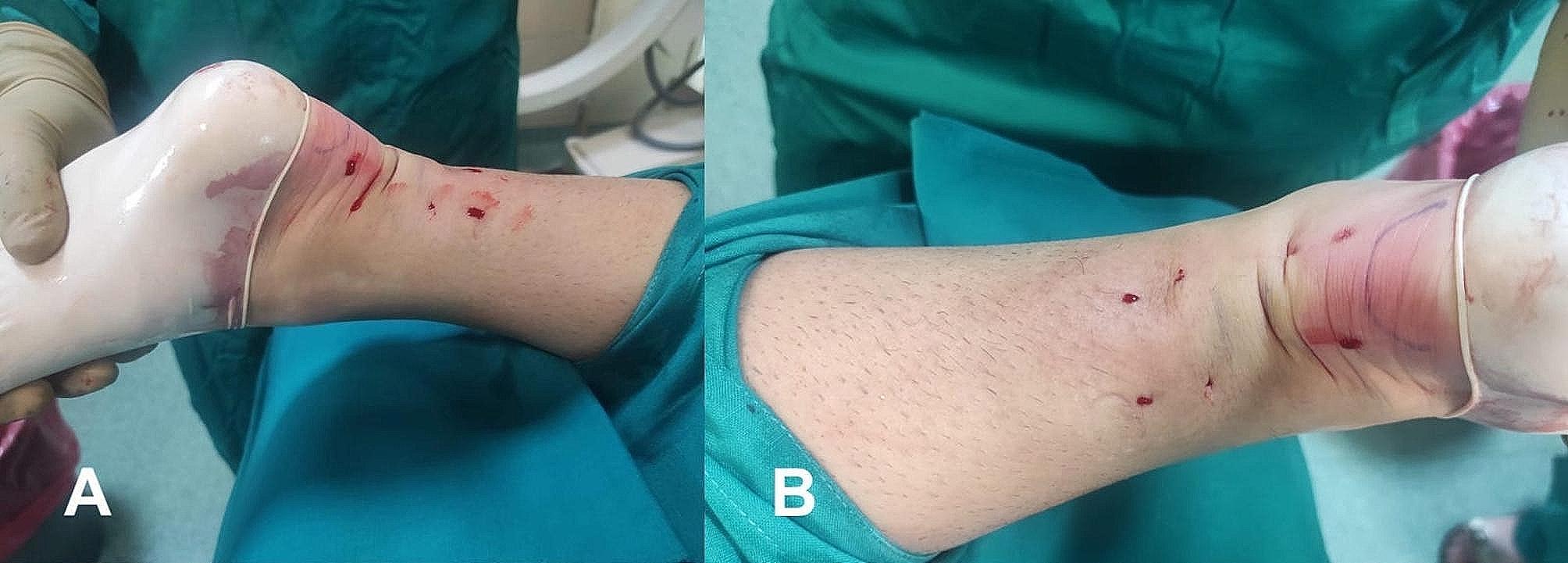
Intraoperative end result of the percutaneous technique. (**A**) Medial view. (**B**) Posterior view

This percutaneous approach allowed us to approximate the broken ends by pulling symmetrically on both sides and simultaneously (Fig. 3D). It was crucial to approximate the torn ends until the defect was no longer palpable, and plantar flexion of the foot likely aided this manoeuvre [8], resulting in the aesthetic outcome shown in Fig. 4A and B.

Open Achilles tendon repair was performed by joining the ends of the tendon using a Krackow locking suture technique. For the Krackow locking suture technique, a resorbable Vicryl suture 2 was utilized.

The choice between percutaneous and open Achilles tendon repair was determined by the attending surgeon based on their knowledge and expertise.

The study included patients who underwent reconstruction of the Achilles tendon through surgical methods (open or percutaneous) or who were managed under the ICD-10 diagnosis S86.0 - Achilles tendon injury. Patients who received conservative treatment for Achilles tendon rupture were excluded from the study.

In both groups (open and percutaneous), patients were administered low-molecular-weight heparin for thromboprophylaxis during their hospital stay. Following hospital discharge, patients were advised to take 150 mg of acetylsalicylic acid daily as ongoing thromboprophylaxis. Thirty minutes prior to surgery, patients in both groups received a single dose of first-generation cephalosporin for antibiotic prophylaxis, dosed at either 1 or 2 g depending on the patient’s weight. For those allergic to penicillin, clindamycin was used as an alternative for antibiotic prophylaxis.

The postoperative care protocol was identical for both patient groups. Initially, the operated limb was immobilized using a dorsal lower leg splint set at 30 degrees of plantar flexion for two weeks. This was followed by a period of two weeks during which the limb was immobilized at 20 degrees of plantar flexion. Subsequently, the splint was removed, and an ankle-foot orthosis was applied while the foot was in a neutral position for the next four weeks. During the first four weeks, while the edorsal lower leg splint was in place, patients were permitted to bear weight with toe-touch activities on the operated limb. In the following four weeks, with the ankle and foot orthosis, full weight bearing while walking with the orthosis was allowed.

The main outcome measures were the presence of complications in patients treated with the surgical method and the number of hospitalization days. A shorter number of hospitalization days indicated a more favorable outcome. All the data from patients who underwent Achilles tendon reconstruction were entered into Microsoft Excel 2020 and statistically analysed using the same program. MedCalc software (v20.110; MedCalc Software, Ostend, Belgium) was used to compare the outcomes between open and percutaneous repair. Qualitative data are presented as absolute and relative numbers. Contingency tables and the chi-square test were used to analyse qualitative data. The associations between variables were evaluated using Spearman’s correlation coefficient. The normality of the distribution of quantitative data was tested using the Kolmogorov‒Smirnov test. Since all the quantitative data were normally distributed, they are presented as medians (quartiles and ranges). The Mann‒Whitney test was used to compare quantitative data between the two groups. The results were interpreted at the significance level of *p* < 0.05. The statistical analysis was performed by the authors of this study.

## Results

**Fig. 5:**
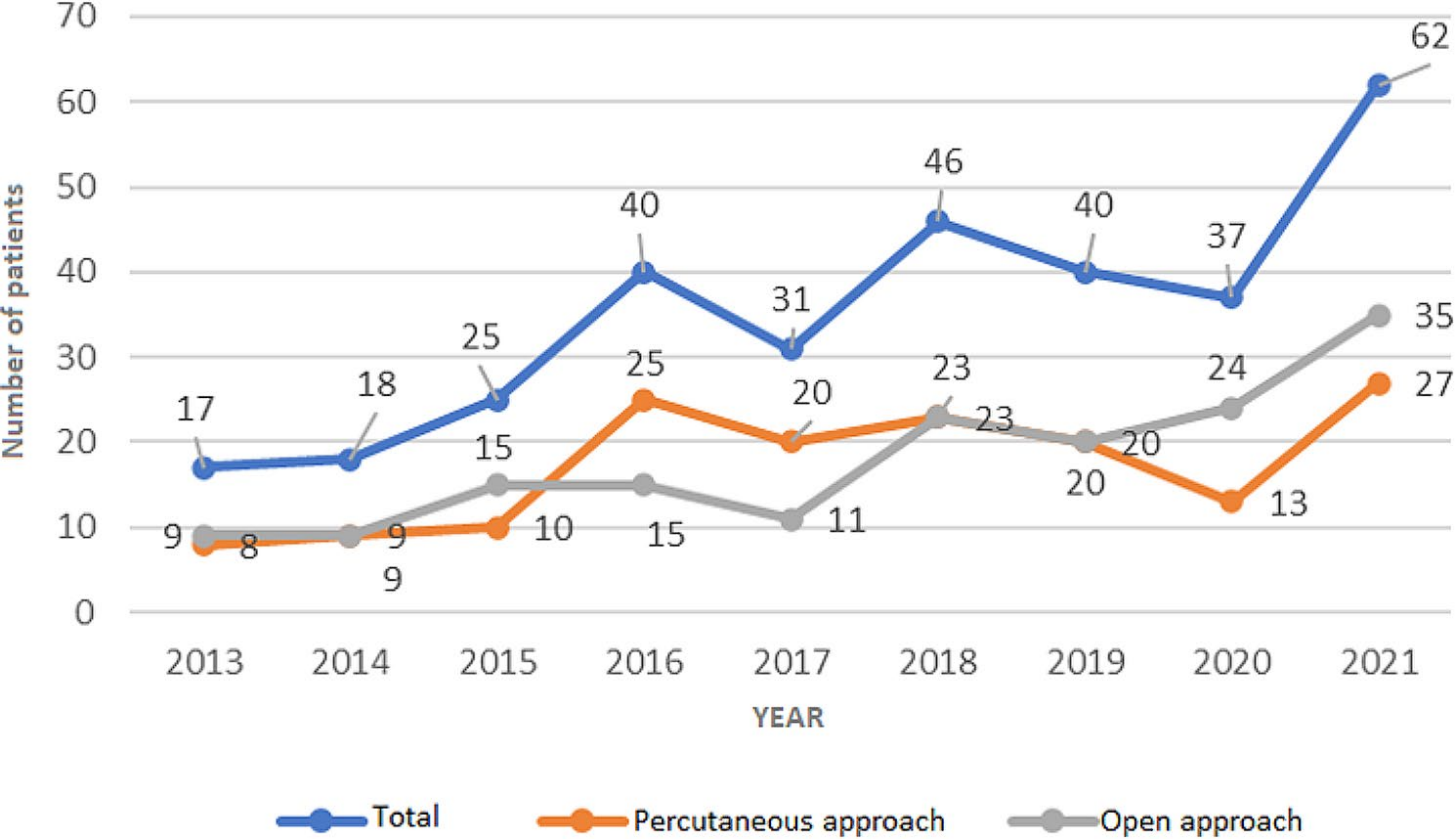
Number of patients treated for Achilles tendon rupture throughout the years of research in total and in relation to the type of procedure

The analysis of the collected data revealed that between 2013 and 2021, 316 patients were treated for Achilles tendon ruptures and were diagnosed with ICD-10 S86.0, an Achilles tendon injury. During this period, two surgical approaches were employed for treating Achilles tendon ruptures: percutaneous in 155 (49%) patients and open in 161 (51%) patients. Figure 5 illustrates the number of surgical procedures performed annually and the approach used to suture the tendon according to the year of the study. A statistically significant increase in surgical procedures was observed over the years of research (Spearman correlation coefficient rho = 0.828; *P* = 0.006).

The sex distribution of the patients revealed that 94% (*n* = 298) were men, while only 6% (*n* = 18) were women. The median age of the patients at the time of surgery was 42 years (Q1-Q3: 35–52; min–max: 18–79 years). For men, the median age was 42 years (Q1-Q3: 35–52; min–max: 18–79 years), and for women, the median age was 41.5 years (Q1-Q3: 35–52; min–max: 21–73 years). There was no statistically significant difference in age at the time of surgery between men and women (Z = 0.140; *P* = 0.889). These data are presented in Table 1.

**Table 1:** Number (%) of patients by gender and median age (Q1-Q3; min–max) in relation to the type of surgical approach

		Operative approach	
		Percutaneous (*n* = 155)	Open (*n* = 161)
Sex			
	Men	146 (94)	152 (94)
	Women	9 (6)	9 (6)
Age (in years)		41 (35–51;18–74)	43 (35–53;20–79)

**Fig. 6:**
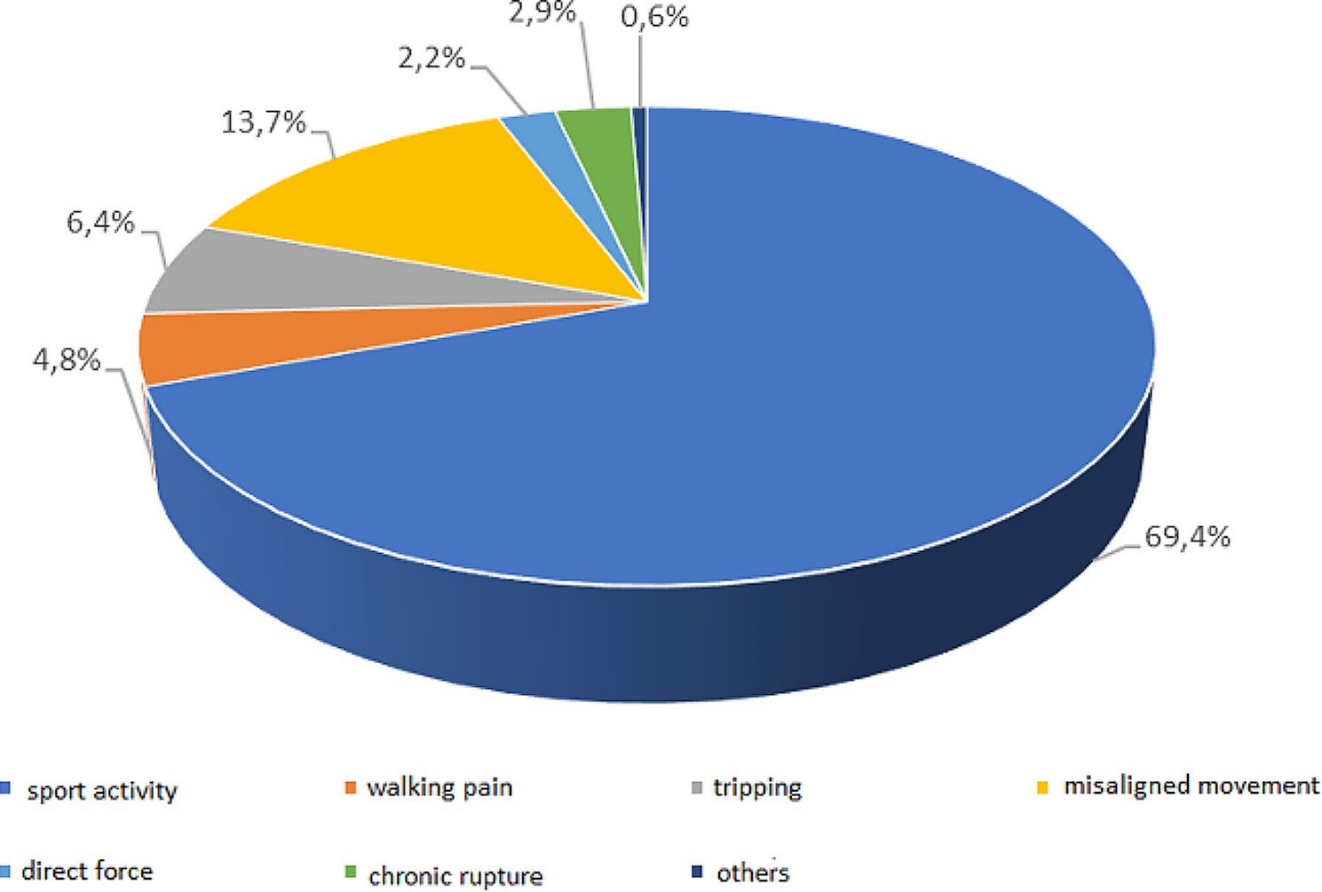
Distribution of patients according to mechanism of injury

The majority of Achilles tendon ruptures occur during physical activity, with a smaller percentage resulting from sudden movements, direct force applied to the Achilles tendon, or chronic rupture. The distribution of injury mechanisms is illustrated in Fig. 6.

Table 2 presents the clinical data of the patients in relation to the type of surgery. The distribution of patients based on the injured leg (left, right) did not significantly differ (χ2 = 0.818; *P* = 0.366). According to the available data, only 8 patients experienced rerupture, 5 from the percutaneous access group and 3 from the open access group. Additionally, 10 patients developed infections, all of whom were within the open approach group. No sural nerve injuries were recorded in the study population. During the observation period, no statistically significant difference was found in favor of one method over the other in terms of sural nerve injury (*p* = 1). A statistically significant difference was observed in the number of infections between the percutaneous approach and the open approach (χ2 = 0.8; *P* = 0.005). However, the small absolute number of patients with rerupture in both groups and the low proportion of patients with this complication relative to the study population size unfortunately preclude an adequate statistical comparison between the two groups in this respect. In other words, a statistically significant advantage of percutaneous repair over open repair for treating Achilles tendon ruptures could not be established during the observed period. The median hospitalization duration was 4 days longer with the open approach than with the percutaneous approach (Z = 14.2; *P* < 0.001). The median distance of the rupture site from the insertion was 1 cm greater in the open approach group than in the percutaneous approach group (Z = 3.98; *P* < 0.001).

**Table 2:** Correlation of the investigated variables with the type of surgery

			Operative approach		*P*
		Total (*n* = 316)	Percutaneous (*n* = 155)	Open (*n* = 161)	
Leg					0,366*
	Left	160 (51%)	83 (53%)	77 (48%)	
	Right	156 (49%)	72 (47%)	84 (52%)	
Rerupture	No	308 (97)	150 (97)	158 (98)	
	Yes	8 (3)	5(3)	3 (2)	
Infection	No	306 (97)	155 (100)	151 (94)	0,005*
	Yes	10 (3)	0	10 (6)	
Suralis injury	No	316 (100)	155 (100)	161 (100)	**1**
	Yes	0	0	0	
Insertion distance (cm)	Median (Q1-Q3;min–max)	5,5 (4,5–7; 1,2–11)	5(4–6; 1,2–8,5)	6 (5–7,5; 2–11)	**< 0,001†**
Time in hospital (days)	Median (Q1-Q3;min–max)	4 (2–7; 0–17)	2 (2–3;0–8)	6 (5–9;2–17)	**< 0,001†**

*χ2 test; †Mann‒Whitney U test

## Discussion

The substantial increase in Achilles tendon ruptures over the last decade, driven by the growing number of recreational athletes, has led to significant progress and innovations in the treatment of these injuries. The multitude of treatment options and the absence of a unanimous consensus within the global scientific community have left ample room for further discussions, disagreements, and research.

This study investigated the surgical treatment of Achilles tendon ruptures using one of two approaches (percutaneous or open) and evaluated the success of the percutaneous approach for such injuries. Patient data were exclusively obtained by reviewing medical records in the database. Based on the available data, conclusions were drawn about operative complications (postoperative infections, reruptures, sural nerve injuries) and the number of hospitalization days following surgery. A comparison of the two approaches in this research showed that the number of infections was significantly lower in the percutaneous repair group than in the open repair group (*p* = 0.005). No statistically significant difference was found regarding sural nerve injury (*p* = 1). Due to the small number of patients with reruptures in both groups, an adequate statistical analysis of the two examined groups was not possible, and the advantage of one method over the other could not be established. A statistically significant advantage of percutaneous repair over open repair was established in terms of hospitalization days following surgery (*p* < 0.001). The primary outcomes of this research demonstrated the high success rate of the percutaneous approach as a surgical treatment for Achilles tendon ruptures.

The American Academy of Orthopedic Surgeons’ guidelines for the treatment of Achilles tendon ruptures outline various parameters for comparing different treatment modalities [9]. Patients undergoing percutaneous suturing of Achilles tendon ruptures exhibit greater satisfaction and improved mental health outcomes due to shorter hospital stays and quicker return to sports. However, there was no statistically significant difference between percutaneous and open methods in terms of returning to physical activity. In terms of surgical complications, there was no statistically significant difference between the two methods, which contrasts with the findings of this study regarding infection rates [10].

Jones et al., in their Cochrane Collaboration review article, examined 14 studies comparing the percutaneous method to the open approach involving 1085 patients. They concluded that there was no statistically significant difference between the two methods in terms of rerupture rates but found a significant difference regarding infection rates [11]. These results align with the present research. The authors also found a statistically significant advantage for the percutaneous method when considering patient satisfaction based on recovery time [11]. While the current research did not directly focus on the duration of the operation or the time to return to physical activity, it revealed a statistically significant advantage of the percutaneous method over the open method in terms of shorter hospital stays.

Yang et al.‘s meta-analysis, based on 12 studies with 815 patients, demonstrated no statistically significant difference in rerupture rates or sural nerve injury rates between percutaneous and open approaches. However, they confirmed the superiority of the percutaneous approach, as it was associated with a significantly lower rate of infection and a shorter duration of operation [12]. These findings are consistent with the current research.

Rozis et al.‘s study yielded similar results, and they also investigated the time to return to physical activity, finding no significant advantage for either method [13]. Although the current research did not examine these specific parameters, they offer valuable directions for future investigations, which could further contribute to the understanding and comparison of percutaneous and open approaches for treating Achilles tendon rupture.

In 2015, Čukelj et al. published a study involving 90 patients and demonstrated that the return to sports activities was twice as fast for patients who underwent percutaneous repair than for those who underwent open repair. They also found that the percutaneous repair group had no postoperative infections or reruptures of the Achilles tendon [14]. Similarly, Čretnik et al. reported a significantly lower number of postoperative complications in the percutaneous repair group than in the open repair group [8], which aligns with the findings of this study.

Although this study did not investigate functional or biomechanical differences between the two surgical approaches, prior research has attempted to address this question. For a long time, the prevailing opinion was that open surgery results in better postoperative outcomes. However, Lazaroni et al.‘s study revealed no significant difference in isokinetic results through mechanical testing between patients who underwent open and percutaneous repair. Additionally, no significant difference was observed in the loss of circumference between operated and nonoperated legs for either approach [15].

Regarding functional outcomes, both Karabinas and Clanton concluded in their studies that the two surgical techniques produced similar results. Clanton’s study noted that patients treated with a percutaneous approach might require longer postoperative protection to allow for biological healing and to prevent early repair elongation and potential gapping between the healing tendon ends [16]. On the other hand, Karabinas et al. reported that postoperative cosmetic appearance and patient satisfaction were greater in the percutaneous surgery group [17].

Taglialavoro et al. conducted a comparative analysis of two percutaneous techniques: the Ma and Griffith methods and the Tenolig method. In the Ma and Griffith approach, the postoperative regimen involved applying a below-knee cast with the ankle positioned in plantar flexion for 30 days. This was followed by a second cast, in which the foot was positioned in neutral alignment for an additional 20 days. Conversely, the postoperative protocol for the Tenolig technique entailed plantar flexion through a below-knee cast with the ankle for 21 days. Subsequently, from the 22nd to the 43rd day, patients were fitted with an articulated walking boot [18].

This study implemented a similar postoperative protocol. Initially, a dorsal lower leg splint was set at 30 degrees of plantar flexion for the first two weeks. For the subsequent two weeks, the splint was adjusted to 20 degrees of plantar flexion. The final phase, lasting four weeks, involved the use of an ankle-foot orthosis set in a neutral position.

The main limitation of this study is its retrospective design and reliance on medical history data, which makes it impossible to compare all treatment outcomes that are functionally significant, such as the time to return to activity, patient satisfaction, and quality of life after surgery. Additionally, the study is limited by its nonrandomized approach for choosing the surgical technique, its focus on results from just one institution, and the lack of both prospective tracking and comparative analysis of functional outcomes. To confirm the results of this research, a multicentre prospective randomized study with a larger number of patients monitored over a longer period of time is necessary.

## Conclusions

This study demonstrated that the main disadvantages of open repair are a greater percentage of infections and longer hospitalization time, resulting in a slight advantage for percutaneous repair. Neither technique significantly increased the risk of rerupture, and the major and most common complication of percutaneous repair, sural nerve injury, was not observed. Therefore, percutaneous repair appears to be a safe method with excellent clinical outcomes.

## Data Availability

The datasets used and/or analysed during the current study are available from the corresponding author upon reasonable request.

## Abbreviations

ICD-10: International Classification of Diseases, Tenth Revision
